# Factors associated with antiretroviral therapy adherence among adolescents living with HIV in the era of isoniazid preventive therapy as part of HIV care

**DOI:** 10.1371/journal.pgph.0000418

**Published:** 2022-06-02

**Authors:** Jimmy Ba Villiera, Hilary Katsabola, Menard Bvumbwe, Joseph Mhango, Justice Khosa, Allison Silverstein, Alinane Linda Nyondo-Mipando

**Affiliations:** 1 Baylor College of Medicine Children’s Foundation, Lilongwe, Malawi; 2 St John of God Hospitaller Services, Lilongwe, Malawi; 3 Texas Children’s Hospital, Houston, Texas, United States of America; 4 Department of Health Systems and Policy, School of Public Health and Family Medicine, College of Medicine, Blantyre, Malawi; Zuckerberg San Francisco General Hospital and Trauma Center, UNITED STATES

## Abstract

High levels of adherence to antiretroviral therapy (ART) are essential to promoting viral suppression and consequential good treatment outcomes. Adolescents living with HIV (ALHIV) in sub-Saharan Africa are less adherent to ART compared to adults, leading to lower rates of viral suppression and immunological recovery. We conducted a mixed-method study utilizing a convergent parallel approach to explore factors associated with ART adherence among ALHIV in the era of isoniazid preventive therapy (IPT) as part of HIV care. The quantitative data were collected from patient records from the period between 1 February 2017 and 31 January 2018 (6 months before and after IPT introduction), while qualitative data was collected from purposively selected patients and healthcare workers by in-depth interviews through a pretested interview guide. A total of 385 patient records (age 10–19 years) were analyzed in the two time periods, while 16 ALHIV (age 10–19 years) and three healthcare workers directly involved in adolescent care were interviewed. Quantitative data utilized logistic regression to measure the strength of association between IPT addition and ART adherence, whereas, qualitative data were analyzed using a thematic analysis approach. The mean age of participants in the quantitative section was 14.3 years (SD 2.7) and 178 were females, while the median age for adolescents interviewed was 14 (IQR 12–17) and 8 were females. Overall, we found an interaction of factors that influenced ART adherence. Added pill burden, on its own, did not affect ART adherence. Commonly reported factors that led to poor adherence were lack of status disclosure within the family, school pressure, and inadequate support from guardians and parents. According to retrospective patient records, complex ART regimens also worsened adherence (*p* = 0.0462). ART regimen was independently associated with adherence (OR 2.11 95% CI 0.97–4.53). Being on ART for a longer duration, enrolment into teen clubs, clinical psychosocial support, and self-reinforcement techniques were suggested to improve ART adherence. The interplay of multiple factors leads to poor rates of adherence. The introduction of IPT to ART packages may not independently affect ART adherence. Readily available psychosocial services and the presence of peer and guardian support is critical to optimal ART adherence. There is a need for ART centers that provide HIV care to adolescents to consider integrating psychosocial and other youth-friendly services into day-to-day clinic operations.

## Introduction

Globally, 1.75 million adolescents are living with HIV (ALHIV) and 1.5 million of them live in sub-Saharan Africa (SSA) [1]. Although AIDS-related deaths have reduced in other groups, they have increased by 45% amongst adolescents aged 10–19 [2]. There is a need to achieve and maintain high levels of medication adherence to attain the full benefits of antiretroviral therapy (ART). Poor adherence leads to viral replication, disease progression, and drug resistance which affects the health outcomes in a patient [3–5].

Adolescents are experiencing disproportionately poor ART outcomes compared to adults, including higher rates of mortality, loss to follow-up, and lower rates of virologic suppression compared to other age categories [6]. Worldwide, adherence rates for adolescents and young adults vary from 52% in North America to 62% in Europe and South America, to 84% in Africa and Asia [3, 7]. When compared to adults, 6 months adherence rates among adolescents living with HIV (ALHIV) in sub-Saharan Africa are low with only 20% achieving 100% ART adherence while over 40% of adults achieve 100% ART adherence [8].

The personal level factors that enhance ART adherence include positive results from the treatment [9], faith in treatment [10], and improved understanding of the disease process [11]. Likewise, improvement in CD4 count and clinical conditions is highly associated with good adherence [9]. ALHIV who witness clinical improvement after starting ART are motivated to be adherent to medication [12]. The motivation to adhere to medication is also high when peers living with HIV who are on ART exhibit an improved health status [11]. The provision of appropriate education and motivation from the doctors, reduction in pill burden, peer and community support further contribute to improved adherence patterns [9, 11]. Evidence suggests that ALHIV who strongly believe in ART lead a disciplined lifestyle that facilitates good adherence and this is strengthened by daily reminders such as the use of alarms [9, 13, 14]. Alarms are popular as a tool for tracking adherence to ART secondary to the widespread availability of cell phones thereby making it easier for ALHIV to keep up with ART adherence [10, 15].

The personal level factors that affect adherence negatively in adolescence include knowledge, attitudes, beliefs, perceptions, and expectations [16]. Patients’ knowledge and beliefs about their illness, the motivation to manage it, expectations regarding the outcome of the treatment, and consequences of poor adherence interact to influence one’s behavior concerning adherence [12, 16, 17]. Therapy-related factors such as complexity of the regimens, pill burden, side effects, duration of treatment, previous treatment failures, frequent treatment changes, and immediacy of beneficial effects further contribute to medication non-adherence during the adolescent period [15, 16, 18].

Pill-burden is particularly important as some adolescents have associated it with potential damage to the body and a higher risk of drug intolerance [13]. Additionally, ALHIV who take many pills per day find it cumbersome leading to suboptimal adherence [11]. The addition of isoniazid preventive therapy (IPT) to the ART package further worsens the pill burden borne by ALHIV. Evidence indicates that isoniazid is effective in reducing Tuberculosis (TB) cases amongst people living with HIV (PLHIV) [19, 20]. However, data is limited on the effects of the added pill burden on overall ART adherence amongst adolescents. While there have been studies assessing factors associated with ART adherence among ALHIV [12, 18, 21, 22], there is a paucity of data on the effects of the addition of IPT on overall adherence to ART in adolescents living in TB and HIV high burden communities [23].

Despite ALHIV being at risk of contracting TB, especially in Southern Africa which is an area with the highest TB prevalence, there is limited data on the effects of IPT implementation on overall ART adherence among ALHIV [24]. Furthermore, few studies have examined the importance of adolescent-specific interventions to improve ART adherence in southern Africa. The unavailability of such data prevents the development of adolescent-friendly interventions when designing new treatments to address adherence challenges and improve treatment outcomes.

Malawi is a landlocked country located in southern Africa and ranks among the poorest countries in the world by annual GDP. The HIV prevalence is 8.8%, and there are an estimated 83,000 ALHIV, making Malawi one of the top countries with the highest number of ALHIV [25, 26]. In 2016, Malawi’s ministry of health adopted and implemented an IPT policy for all HIV-infected patients on ART in five TB high burden districts to reduce the incidence of the disease, estimated at 146 cases per 100,000 population in 2019; with a 48% TB/HIV co-infection rate [27–29]. This study explored factors associated with ART adherence among adolescents in an era of IPT as part of HIV care at a tertiary paediatric HIV facility in the central region of Malawi.

## Methodology

### Study design

We employed a convergent parallel study design utilizing both quantitative and qualitative approaches to assess the effects of IPT and other factors on overall medication adherence among HIV-infected adolescents [30]. The quantitative component involved a retrospective review of medical records of ALHIV aged between 10–19 years, receiving care at the centre for at least 6 months before and after the introduction of IPT. The qualitative section utilized a phenomenological approach comprising in-depth interviews with ALHIV in care at the centre and key informant interviews with healthcare workers who provide care to the adolescents. We selected a convergent parallel design because it allowed the researchers to corroborate and support the results relative to the same phenomenon and to ameliorate internal and external validity [30, 31]. While quantitative approaches measured the strength of association between our variables of interest, the qualitative data offered a holistic examination of a person within their natural environment and highlighted patient-related factors that are associated with poor adherence [31].

### Study setting

This study was conducted at Baylor Malawi which operates the Baylor College of Medicine- Abbott Fund Children’s Foundation—Clinical Centre of Excellence (BCMCFM-COE), situated within Kamuzu Central Hospital (KCH) in Lilongwe, Malawi. BCMCFM-COE is a stand-alone paediatric referral clinic that serves as an outpatient paediatric HIV clinic for KCH and a paediatric referral centre for the entire country with most patients coming from the central and northern region of Malawi. BCMCFM-COE is the largest provider of paediatric HIV care and treatment services in the country. The centre has the largest cohort of paediatric HIV-infected children in Malawi and enrols HIV-exposed and infected children from 6 weeks to 18 years of age. The centre started offering HIV services in 2005 and currently serves over 3500 active patients mostly from the central and northern regions of Malawi. The clinic has no age-specific discharge criteria as such the clinic sees clients who are older than 18 years as long as they were under the age of 18 at the time of enrolment. BCMCFM-COE supports other government facilities with physician and mentorship services to improve HIV care in the country. The clinic is manned by paediatricians, clinical officers, and nurses and on average, 90 clients are attended to daily. The overall adherence rate for the clinic is between 90% to 100% [32]. Unlike other ART clinics, clinicians and nurses at BCMCFM-COE are certified to prescribe second-line regimens of ARVs in Malawi as well as management of advanced HIV disease.

#### Recruitment, sampling, and data collection

The study population was a cohort of male and female adolescents who were 10 to 19 years of age. We enrolled adolescents that were on ART, on IPT (patient must have started according to national guidelines), known age at the start of IPT initiation, had at least 2 medication refill appointments within the pre-and post-implementation periods and had continued with IPT for 6 months following initiation [28, 29]. We excluded adolescents that were initiated on IPT but were lost to follow-up or discontinued IPT—either self-directed or as per the medical provider’s guidance.

We made certain that participants’ HIV status was fully disclosed during the qualitative component. Disclosure status was verbally verified before the in-depth interviews by asking an adolescent why they take medications daily. Furthermore, we used extreme deviant case sampling by including participants that reported either extremely good adherence (pill count between 95%-100%), or extremely poor adherence (pill count less than 75% or above 125%), and finally, adolescents who were also included in the quantitative cohort and either (1) attend Teen Club or (2) still follow at BCMCFM-COE. The study only included participants on the first-line and second-line ART regimens. This was the case because first and second-line ART regimens are relatively easy to take compared to third-line or non-standard regimens.

Healthcare workers who have worked with ALHIV for at least five years were included as study participants. To improve the variability of Healthcare worker responses, clinicians, nurses, and psychosocial workers were offered the opportunity to take part in the study.

For the quantitative component of the study, we used the following formula n = *Z^2^ P(1-p) / d^2^* [33, 34] to calculate sample size (n = sample size, *p* = expected proportion of non-adherent clients, *Z* = confidence interval—statistic corresponding to the level of confidence, *d* = margin of error—corresponding to effect size). Using national adherence rates in adolescents (45%), a total of 385 medical records that met the inclusion criteria were selected for review using a computer-generated simple random selection technique to represent active clients at the facility [4]. For the qualitative component, we followed a purposive sampling technique to select 16 ALHIV and three healthcare providers for in-depth interviews and key informant interviews respectively. The qualitative sample size was based on assertions from Guest *et al*. who stated that by the 12^th^ interview one would have reached saturation. However, in our study, we included 16 participants because of the distribution of the variables of interest in our purposive sampling approach [35]. ALHIV were selected according to their adherence pattern which was determined by physical pill counting or reported pill count. Eight participants that were regarded as very adherent were selected together with eight participants who usually reported suboptimal adherence. Adolescents who met the criteria and were in care at the study site between the period of 1 February 2017 and 31 January 2018 were considered for study participation. Healthcare providers were also purposively selected based on their involvement in the provision of care to the adolescents at the study facility. The non-probability sampling method was deliberately selected to have a sample that contains characters of interest [36].

Quantitative data comprised a retrospective review of prospectively collected data of ALHIV who were in care between 1 February 2017 and 31 January 2018. A checklist containing variables of interest; age, sex, ART regimen, IPT, and Cotrimoxazole preventive therapy (CPT) status, disclosure status, education level, and religion was used to extract medical records from Electronic Medical Records (EMR). The study site uses EMRx which has a variety of features for the clinic, laboratory, psychosocial, and pharmacy, among others, to record medical records at each hospital visit. Some of the participants’ records selected for review in pre IPT period were not included in the post IPT review if they did not satisfy the inclusion criteria.

Qualitative data was collected through in-depth interviews and key informant interviews using semi-structured interview guides. Participants were approached during teen clubs where they were asked to participate in the study after accessing all the services they came to the clinic for. Healthcare providers were interviewed as key informants as they have been involved in the care of ALHIV and have a vast knowledge of adolescent behaviors towards medication. We deliberately interviewed the participants after accessing services and health care workers at their convenient time to realize undivided attention during interviews and to avoid disruption of work respectively. Once a participant signed a consent form a semi-structured interview guide was used to ask questions. The interviews were audio-recorded to capture every detail.

The interview guides were in both Chichewa and English and were utilized according to the language the participant understands and preferred. The interview guides included questions about medication knowledge, facilitators and barriers to adherence to ART, and general perceptions of IPT’s inclusion in the HIV care package. The interview guide for healthcare workers further included questions about the general training and healthcare knowledge on IPT and its side effects. Interviews were conducted in a secure, closed room, and participants were identified by assigned codes as a measure of safeguarding their privacy.

To minimize the threats to the credibility of the study, the investigator used member checks and triangulation approaches [37, 38]. Credibility refers to the confidence that can be placed in the truth of the research findings by establishing whether research findings represent plausible information drawn from the participants’ original data [39, 40]. During the interviews, the information given by the participants was summarized before asking them to verify if the captured information was accurate. Member-checking ensured that the information collected is shared with participants so that intentions are clarified and errors if any, are corrected. We also mixed the data from the interviews with the secondary data to enhance cross-data validity checks [37]. Furthermore, the PI who is an experienced HIV clinician also engaged and trained an additional experienced clinician to help with face-to-face interviews while maintaining strict confidentiality throughout the process. Researchers who were involved in data collection took notes about participants’ comments and researchers’ thoughts during interviews. Additionally, reflective notes from the data were recorded soon after the interviews (memoing). This practice was vital to the process of bracketing and reflexivity in mitigating the potentially deleterious effects of unacknowledged preconceptions related to the research and thereby increasing the rigor of the research [41, 42]. The PI verified all the transcripts that were translated from Chichewa to English for completeness and relevance. We also triangulated the analysis approach by using multiple data analysts. A second analyst who was not involved in data collection independently reviewed the interview data. Any gaps or blind spots were discussed with the PI, and the aggregated results were documented. Using multiple investigators throughout data collection and analysis reduced the risk of bias caused by a single person doing all the tasks.

### Data analysis

#### Quantitative data

Quantitative data that was extracted from EMR was cleaned using an excel package. To ensure the confidentiality of the participants, we assigned unique numbers to each participants’ record. Both data sets were inspected for completeness before actual analysis. The data in Excel was then exported to R Studio (Ver. 1.3.959) for analysis.

The primary outcome variable for this study was ART adherence and it was measured as a dichotomous variable. All continuous variables such as the age of participants were analyzed using frequency distribution graphs. Descriptive statistical methods such as means, standard deviation, and proportion were generated to further examine our population. Furthermore, statistical analyses using 2-group and regression analysis were executed to measure the association of pill burden, gender, ART regimen, HIV disclosure status, and ART adherence. Variables of interest namely; ART regimen, gender, disclosure status, and IPT status were included in the final multivariate analysis using a forward stepwise approach, regardless of whether they were statistically significant or not in the univariate analyses. The factors were included in the logistic regression model because theoretically these variables are known to interact with each other in adolescent health which can impact adherence [1, 2]. A two-group test using χ^2^ was executed to make crude comparisons of ART adherence before and after IPT introduction and its associated factors with a *p*-value set at 0.05. Logistic regression analysis was used to quantify the association between IPT inclusion and ART adherence.

ART adherence was defined as taking at least 95% of the doses (and no more than 105% from losses) at the prescribed interval and within the refill period to prevent HIV drug resistance [28]. In situations where ART pills were not brought to the clinic for pill-counting, missing no more than 3 doses in a month was also defined as good adherence. We favored logistic regression in this study because our outcome of interest was dichotomous. Furthermore, it allowed for statistical control of known confounders such as concurrent adherence reinforcement interventions; this is a particularly important factor because we used a before and after study design.

#### Qualitative data

We employed a thematic analysis of the data collected through in-depth interviews and key informant interviews as suggested by Braun and Clarke [43]. All audio recordings were translated and transcribed verbatim simultaneously if the interview was not done in English. All interviews conducted in English were immediately transcribed. Chichewa audios were transcribed straight into English by a transcriber conversant in English and Chichewa and were verified by the PI against the audios to ascertain quality and correctness. The researchers manually analyzed the data. The researchers initiated the process of immersion and familiarization with the data while conducting the interviews, and later on, read and re-read the transcripts multiple times to understand the depth of the content. We inductively and deductively coded the data. The inductive codes were drawn from the collected data while theoretical codes were deduced from the interview guide and study objectives. We identified abstract themes from the coded text segments that formed a similar coherent familiar pattern, which was further organized to develop themes. Then the researchers reviewed and refined potential themes to match the rest of the data, where the themes were not complete or did not match the rest of the data or objectives, additional coding was done. The thematic analysis allowed the researchers to interpret and summarize the themes for a report to be produced. Both data types were utilized in a narrative integration manner where we used a weaving approach to present data on a concept by concept basis [44].

### Ethics approval and consent to participate

Ethical approval to conduct the study was granted by the College of Medicine Research Ethics Committee (COMREC certificate number P.11/19/2857), and permission was obtained from BCMCFM-COE. Furthermore, informed consent was sought from all ALHIV before they participated in the study. The informed consent/assent forms were in both English and Chichewa (primary language). Participants aged 18 years and above gave informed consent independently. Participants aged below 18years assented to the study, and their parents/guardians signed the guardian informed consent form. Verbal consent was sought from all health workers before the interviews. Throughout the study, participants were identified by a unique number to conceal their identities. Participants were informed that study participation was voluntary and they had the right to withdraw at any point if they felt uncomfortable or they simply so wished.

## Results

### Quantitative data

#### Characteristics of the quantitative sample

The social demographic characteristics of the participants based on the data extracted from EMR from 1 February 2017 to 31 January 2018 are summarized in Table 1 below. There were a total of 385 participants, mean age of 14.3 years (SD = 2.7). One hundred and ninety-three participants had their records analyzed before the introduction of IPT while 192 had records analyzed after the introduction of IPT into the ART package. Ninety-one percent of participants (n = 349) were on first-line ART at the time of the study, while the rest were on second-line ART.

**Table 1:** Summary of sample demographics.

Variable	N	%
Gender		
Male	178	46.2
Female	207	53.8
Education		
Primary	288	74.8
Secondary	97	25.2
ART regimen		
1^st^ Line	349	90.6
2^nd^ Line	36	9.4
Average adherence		
Optimal	270	70.1
Suboptimal	115	29.9
Disclosure status		
None	12	3.1
Partial	59	15.3
Full	314	81.6

### Qualitative data

#### Demographic characteristics of study participants

We interviewed 16 adolescents aged between 10 and 18 years who are living with HIV and were in care at BCMCFM-COE and three health workers. The median age of the adolescent was 14 with an interquartile range of five (12–17). Out of the 16 adolescents interviewed, eight were females. Eight of the adolescents reported good ART adherence at least 3 months before the interview and the rest reported suboptimal adherences. All adolescents attended school but only five were in secondary school. Only six adolescents were living with non-biological caregivers and all adolescents belonged to religious affiliation, five of whom were Muslims. All adolescents were on IPT at the time of the interview and 13 had full disclosure on their HIV status-completed as per guidelines while three had completed a partial disclosure of their HIV status. Partial disclosure refers to status disclosure in which the client becomes aware of an existing health problem without necessarily knowing the actual condition. It acts as a preparatory step in HIV status disclosure for children. Table 2 summarises the demographic characteristics of participants who were interviewed.

**Table 2:** Demographic profile of participants who were interviewed.

Variable	N	%
**Adolescents**		
Gender		
Male	8	50
Age		
<15	7	44
15–19	9	56
Religion		
Christians	11	69
Muslims	5	31
Education		
Primary	11	69
Secondary	5	31
Guardian Type		
Biological parents	10	62
Others	6	38
Average Adherence		
Optimal	8	50
Suboptimal	8	50

Of the three health care workers interviewed (Key informant interviews), one was a senior clinician and ART provider, one was an adolescent nurse and the last one was a social worker. All the health workers had worked with adolescents for over five years and were directly involved in the care of adolescents both in the clinic and teen club activities. The social worker was particularly involved in developing curriculums for teen club lessons on life skills and psychosocial issues. She was also responsible for handling any psychosocial issues affecting adolescents on ART, including planning and carrying out disclosure and adherence counseling sessions for adolescents.

### Barriers to antiretroviral therapy adherence

#### Factors that affect adherence

In general, the non-adherence rate to ART was 27.5% in the pre IPT period, and 32.3% after IPT was added to the ART package. On factors associated with ART adherence before IPT, only *ART regimen* (*p*-value = 0.0462) was associated with ART adherence leaving out *gender*, *(p*-value = 0.10) and *disclosure status* (*p*-value = 0.55). *ART regimen* (*p*-value = 0.0537) was also associated with adherence after IPT was introduced (Tables 3 and 4).

**Table 3:** Bivariate analysis of factors associated with ART adherence before and after added pill burden (IPT introduction).

Factor	Category	Before IPT introduction			After IPT introduction		
		Adherent n(%) N = 140	Non-adherent n(%) N = 53	P-value (χ^2^)	Adherent n(%) N = 130	Non-adherent n(%) N = 62	P-value (χ^2^)
HIV status disclosure	Yes	116 (82.9)	42 (79.2)	0.37	107 (82.3)	49 (79.1)	0.84
	Partial	21 (15)	11 (20.8)		17 (13.1)	10 (16.1)	
	No	3 (2.1)	0 (0)		6 (4.6)	3 (4.8)	
Gender	Male	60 (42.9)	21 (39.6)	0.68	61 (46.9)	36 (58.1)	0.14
	Female	80 (59.1)	32 (60.4)		61 (53.1)	26 (41.9)	
ART regimen	First line	128 (91.4)	47 (88.7)	0.0462	121 (93.1)	53 (85.5)	0.09
	Second line	12 (8.6)	6 (11.3)		9 (6.9)	9 (14.5)	

**Table 4:** Multivariate logistic regression model of factors associated with ART adherence.

Factor	Category	Standard Error.	Odds ratio	95% Conf. Interval	p-value
Gender	Male	0.23198	1.1977051	0.75–1.88	0.43
	Female				
ART regimen	2ndline	0.38852	2.1161580	0.97–4.53	0.0537
	1^st^ line				
HIV Status Disclosure	Yes	0.73690	1.4258939	0.36–7.15	0.63
	No	0.75194	2.1956099	0.54–11.2	0.29
	_Cons	0.93506	0.1281719	0.018–0.7	0.0280

Multivariate analysis of factors associated with adherence in adolescents showed that only the ART regimen was somewhat associated with adherence. OR 2.11, 95% CI 0.97–4.53, *p*-value *=* 0.0537. (see Table 4). However, since the 95% confidence interval includes a null value, the difference in ART regimen doesn’t affect ART adherence. Consequently, the ART regimen was not associated with ART adherence in adolescents living with HIV/AIDS at Baylor Children’s Centre of Excellence.

#### Adherence before and after IPT introduction (Added pill burden)

Overall, females were more adherent to ART than males at 72% (149/207) versus 68% (121/178) in the period of study (6 months before, and 6months after IPT introduction). However, the difference in adherence between males and females was not statistically significant (χ^2^ = 0.7321, *p*-value = 0.39). Seventy-one percent (249/349) of clients on the first-line regimen reported good adherence while 58% (21/36) of clients on the second-line had acceptable adherence. Although ART adherence was different as per the ART regimen, the overall difference was not statistically significant (*p-*value = 0.10). After IPT was introduced, female participants maintained optimal adherence 53.1% (69/130), than males 46.9% (61/130). However, the difference in ART adherence between males and females after added pill burden was not statistically significant (*p*-value = 0.392).

*Pill burden*. Participants stated the addition of preventive therapies within HIV management such as Isoniazid for prevention of TB and Cotrimoxazole for prevention of Pneumonia and some diarrheal diseases significantly increased the number of tablets. The majority of participants stated that they regarded IPT as a drug outside the ART package and considered it as not important. Therefore, IPT added an unnecessary pill burden. The participants narrated that:

*Yea, IPT somehow affected the number of pills to take in a day*, *but not necessarily on ART*, *because most of the time I would take ART and skip IPT*. *Male 4*

*Sometimes, I could feel I had many tablets to take but it was still manageable because I had about 4 tablets to take at a go*. *However, I got used to the number of tablets so everything was okay*. *Male 2*

Health workers corroborated the adolescents’ view and stated that the introduction of IPT led to adherence problems where adolescents could skip all medications, or at times only adhere to ART and Cotrimoxazole, but skip IPT. This was the case because of the added pill burden coupled with poor client preparation on anticipated side effects and increased number of tablets as explained in the following excerpts:

*There wasn’t much of a change in ART adherence*, *but we realized that most of them were concentrating on ART and CPT and not IPT*. *It looks like when they thought IPT was bothering them due to the pill burden, they only concentrated on the old meds*. *HCW III*

The burden from the additional pills was even worse for adolescents on complex ART regimens as stated by one health worker:

*I feel they did not have the urge to take IPT. It is the case because I feel IPT was implemented in a hurry*. *We didn’t have enough time to prepare them*. *After all, when we introduced it we used to tell them that it helps prevent TB in those whose immunity is low*. *Most of our adolescents are on second-line ART with complex regimens*. *HCW III*

*ART regimen and adherence*. Overall, participants on first-line ART had better adherence (92.2%, 249/349) compared to those on second-line ART (7.8%, 21/36, *p*-value = 0.104). This trend was also true even when the participants were split into before and after IPT cohorts. There was a significant difference in adherence between first and second-line ART regimens in females after IPT was introduced (72.6%, 69/95, p-value = 0.0462).

*Disclosure status and ART adherence*. Eighty-three percent (223/314) of fully disclosed participants showed optimal adherence compared to their non-disclosed counterparts. This difference was not statistically significant when the variables were analyzed as either combined or split cohorts. (*p*-value = 0.55). The bivariate analysis did not show any effect of HIV disclosure status on overall ART adherence.

*Lack of HIV status disclosure*. Participants who live with relatives or friends to whom they have not disclosed their HIV status expressed difficulties in keeping up with ART adherence. This is because, unlike other medications, ARVs have a stigma attached to them. As a result, ALHIV willfully forego medication when in the company of undisclosed acquaintances or relatives. The following excerpts indicate this finding:

*Taking my medications is problematic when I am busy, sometimes when I am among friends it is hard to take my medication too*. *I don’t want them to ask many questions and know about my status*. *Female 7*

HIV status disclosure remains a challenge even among close family members. To save marriages, some guardians prefer to conceal their HIV status or that of their children. One participant stated that her mom categorically indicated that she should never mention her HIV status when her stepdad is around which prompts her to willfully skip her doses whenever her stepdad visits them. This is what she said:

*Having visitors at home or when my stepdad comes home, it becomes an issue to take my medications*. *He [Stepdad] doesn’t know that I am on ARVs and my mom said we should keep it that way*. *Female 3*

Other factors that influenced adherence that were only apparent in the qualitative analysis were as follows:

*Pressure secondary to academic responsibilities*. All the respondents highlighted school activities as one of the barriers to good adherence. Many issues emanated from those participants in boarding schools who stated that keeping up with activities such as daily study periods brought a lot of problems with adherence. For instance, adolescents stated that their evening study periods in school collided with evening ART doses to the extent that most participants could end up missing the doses. Further to this, the quest to take ARVs without schoolmates noticing was a major challenge for most participants. These findings are explained in the following excerpts:

*When I have school work to do and a tight schedule I forget my medication*. *This is usually the case because I have to get up early, prepare for school and I end up forgetting to take my pills*. *Female 4*

One health worker observed that most suboptimal adherence rates are recorded during school sessions for those in boarding schools and at the level of taking national examinations. It was further stated that the lack of monitoring among boarders that are on ART contributes to suboptimal adherence.

*School activities interfere with adherence greatly*. *Even older teens do complain that studies and other activities are quite hard to accommodate adherence. Even going on school holiday has been a very big issue as there is no or little monitoring. Parental support and continued teen club sessions have always been key to good adherence in our school-going teens*. *HCW I*

*Peer pressure*. One health worker affirmed that lack of disclosure is indeed a barrier to achieving optimal ART adherence, and it extends to partners. He further observed that peer pressure among adolescents is an important factor that has the potential to hinder good adherence. Adolescents who engage in love relationships because of pressure from friends are likely to hide their HIV status from partners. This further means sometimes skipping doses when they are close to their lovers for fear of being questioned which has the potential of leading to unintended disclosure.

…*love relationships among adolescents worsen adherence because partners do not usually disclose to each other for fear of losing love partners*. *Peer pressure from friends is also an important barrier*. *When they are interacting some non-adherent peers give tips to friends on pill counting so that they show good adherence at the clinic when they are skipping doses*. *HCW III*

*Poor support*. Adolescents mentioned a lack of parental, peer, or sibling support as a barrier to adherence. Adolescents who stayed with non-supportive parents and peers reported difficulties adhering to medication. This is what they said:

*When I fight with my siblings I get so angry to the extent that I don’t take my meds… “panthawi imeneyo ndimakhala kuti mankhwala ndawataya uko*!*” (At that moment, I don’t want anything to do with my medication)*. *Also when I disagree with my mom it’s the same*, *I don’t take my meds. But these have nothing to do with IPT*. *Male 2*

*When I fight with my siblings they throw my drugs out of the house*. *They are mean to me sometimes, so that affects me. Otherwise, the number of pills never bothers me*. *Male 1*

Health workers stated that non-adherent peers give tips to adherent ones such as pill dumping and pill calculations to show good adherence at the clinic when they are not taking their medications.

*Peer pressure from friends is a negative influence on adolescents*. *When they are interacting some non-adherent adolescents give tips to show good adherence at the clinic*. *HCW III*

### Factors that facilitate adherence to ART

The factors that facilitate adherence to ART are at personal and institutional levels. Personal factors that enhance ART adherence include self-reinforcement techniques and the longer duration of ART.

#### Patient-level factors

*Self-reinforcement techniques*. Participants stated that they fail to take medication due to forgetfulness. To deal with forgetfulness, participants employed several measures for them to remember when to take medicines including the setting of alarms. Phone alarms are commonly used because phones are more widely available than proper clock alarms. Another tactic used by adolescents to remember to take their medicines was the act of keeping medications where one can easily access them. Some participants reported that they kept their medication bottles in their pillowcases or shoes. Participants explained that it is easy to remember to take medication at night once they hear the clanking sound of the ART bottles in the pillowcase. The explanation was similar for those who keep medication in the shoes to remember in the morning before going to school as indicated in the excerpts below:

*I use a calendar and tick it every time I take my meds*, *when I am at school I sleep with my pills so that I should remember in the morning*. *Male 4*

*I use the alarm on my mom’s phone, sometimes when the phone is off, I use a wall clock*. *I also put my medication in my school shoes when all these things are not available so that I take them in the morning before going to school because I keep my medication*. *Female 6*

*The longer duration of ART*. Participants stated that being on ARVs for longer duration makes one more responsible as one becomes fully aware of the need for ARVs. They explained that unlike those who are just starting ART and are heavily reliant on parents, adolescents on ART for a longer duration must have gone through ART and adherence sessions more than once such that they are familiar with adherence issues.

*Being reminded by my parents but also just being responsible*. *I have been on medication for years and I know I have to take my medications every day*. *So I make sure I take my ARVs after all I keep my pills myself*. *Female 6*

#### Health system and social factors

The health system and social factors that facilitate adherence to ART among adolescents include Teen Club services, and peer, guardian, and clinic-based psychosocial support.

*Teen club activities*. It overtly surfaced that teen clubs played a great role in reinforcing adherence among adolescents. The lessons that are delivered at the teen club, peer experiences, the sense of belonging, and the feeling that there are equally other teens on ART who are doing well help adolescents to cope with their HIV status and be adherent to medications. One participant stated that despite the different adherence techniques he uses; the teen club has been instrumental in maintaining good adherence because of the adherence lessons offered. Many participants further explained that the lessons help them not only to remember to take medication but also to take good care of themselves both at home and school. This is what participants said:

*With exposure to a teen club, things are different*. *My adherence used to be poor because I was not exposed to life skills coaches and I was not feeling encouraged. It’s different from the time before I joined the teen club*. *Female 8*

*Further to several techniques we use*, *I think a teen club is one important thing holding us adolescents*. *Because when we come here we end up learning one or two things about how we can look after ourselves, how to take medications, and deal with other important issues such as social stigma at school. When you are here you learn more and make important and good decisions. Male 2*

*Peer*, *guardian*, *and clinic-based support*. Parental/guardian and peer support also emerged as an important facilitator to medication adherence. The majority of participants stated that parental or guardian support was vital to adherence. Participants stated that being reminded of the time to take medication and getting support such as transport money to and from the clinic on refill appointment dates were perceived to be important. One participant observed that being reminded and being shouted at by his guardian whenever he misses doses helps him to be more adherent to his medication. This was further expounded by one participant who stated that he found comfort in the fact that he was not alone taking ARVs in the house because his two siblings were also on ARVs which encouraged him to be adherent to his ARVs.

The following were some of the responses:

*Having relatives who are also taking drugs is very important*. *They remind you*. *Family support through reminders and keeping drugs in places where you can easily see them helps to remind you that you need to take meds. My parents remind me to take my meds when they are taking their meds*. *Female 3*

*Being reminded by my mom and being shouted at is a constant motivation to me*. *I know that if I skip my medication my mom will shout at me and that alone reminds me to take my medication all the time*. *Female 5*

Health workers affirmed the role of psychosocial counseling, peer interaction during teen clubs as well as building rapport with adolescents as key factors that facilitate good adherence. One healthcare worker narrated:

*Well for me, interaction with adolescents is important. They can take our advice because we are in a good relationship with them, Secondly, the fact that we temporarily suspend them from attending teen club when they are non-adherent also helps them to keep up the good work so that they don’t miss Teen Club. And the frequency of refill visits also helps them to be adherent*. *Those who are non-adherent have to come to the clinic frequently*. *HCW III*

## Discussion

Our findings show that the non-adherence rate to ART was 32.3% in the period after IPT was added to the ART package compared to 27.5% in the pre-IPT period. The factors that lead to poor ART adherence among adolescents include the type of ART regimen, lack of status disclosure within the family, school pressure, and inadequate support from guardians and parents. The factors that facilitate adherence to ART among ALHIV are at the personal and health system levels. The personal factors include self-reinforcement techniques and being on ART for a longer duration. The health system factors include the availability of teen club services and clinic psychosocial support.

Our results on the type of ART regimen as a factor leading to suboptimal adherence are similar to previous studies that attributed suboptimal adherence to complex ART regimens that require the intake of multiple pills per day [11]. ALHIV on second-line ART find it difficult to adhere to medication as they have to take drugs twice a day making it easier to skip doses. Additionally, the fear of toxicity from multiple drugs in most second-line regimens negatively affects adherence [12, 18, 21, 45]. It is worth noting that suboptimal ART adherence among ALHIV on second-line ART regimen could likely emanate from pre-existing adherence challenges which made them fail on first-line ART and not necessarily as a result of the addition of IPT. We suggest a deliberate and continuous adherence support strategy in ALHIV on second-line ART for improved adherence. There is a need for Malawi to scale-up psychosocial services and teen club enrolments to support second-line clients to ensure frequent engagement, and peer support where adherence reinforcement techniques can be discussed [46–48].

A lack of HIV status disclosure within families was associated with non-adherence to medication, which is consistent with findings from a previous study that reported that HIV status non-disclosure to a loved one or fear of stigma is a risk factor for non-adherence to HIV medication [8]. As HIV-infected children slowly transition to adulthood, aspects of status disclosure and counseling require support and reinforcement. As they transition, adolescents engage in love relationships which may affect adherence if they are not well prepared [10]. Although HIV status disclosure was reported to be a catalyst for optimal ART adherence during participant interviews, a review of patient records did not show significant differences in adherence regardless of one’s HIV disclosure status (χ^2^ = 1.1679, *p* = 0.55). The contradicting results between qualitative and quantitative sections can be attributed to reporting errors where non-disclosed adolescents may be identified as fully disclosed and vice versa. This is in sharp contrast with findings from studies on the effects of HIV status disclosure on ART adherence done in Uganda, Kenya, and South Africa where they observed a positive effect of status disclosure on ART adherence in ALHIV [17, 49, 50] The current study’s contrary findings may also be associated with advanced HIV care, treatment and support provided at the study site considering its status as a tertiary facility in HIV management.

In our study, we noted that school activities interfered with ART adherence for most ALHIV due to challenges in balancing school pressure and keeping up with ART uptake. Similarly, an earlier study reported that limitations in school attendance were associated with lower odds of non-adherence [51]. Although school pressure affects adherence, limiting school attendance is not a viable option in a typical Malawian setting due to, among others, a lack of resources for homeschooling or hiring private teachers. Therefore, there is a need to prepare school-going ALHIV both physically and psychologically so that they should be able to keep up with adherence while dealing with school demands [17, 52].

Our findings showed that inadequate or lack of peer and guardian support was the most common reason for suboptimal ART adherence among adolescents and this resonates with findings from a Malaysian qualitative study that reiterated that the lack of peer and caregiver support brings about a sense of hopelessness which results in poor adherence among adolescents [11]. Adolescents who never receive caregiver support miss medicines because they feel depressed and overwhelmed [4]. This is an interesting finding because it also emphasizes the need for thorough and targeted disease counseling for a caregiver of adolescents on lifelong treatment. In this instance, we commend the government of Malawi for integrating HIV/AIDS services with other sector programs such as TB, nutrition as well as reproductive and child health [53]. This ensures a family-centered approach to the management of HIV which encourages caregiver support to children and adolescents.

The use of self-reinforcement techniques and being on ART for a longer duration enabled adherence which was also reported in an Ethiopian study that showed that clients who used alarm clocks as reminders were more likely to have optimal ART adherence compared to those who did not [54]. Our finding encourages health workers to teach and emphasize self-reinforcement techniques that have proven to work in enhancing adherence. A longer duration of ART was associated with good adherence which is consistent with findings by Malee *et al*. who reported that HIV-infected children and adolescents who had been on ART for more years had lower odds of non-adherence [51]. This finding is particularly important for healthcare providers and policymakers to make evidence-based decisions on resource allocation. For instance, this finding persuades decision-makers in health services to invest more in the training of providers so that they can competently prepare and support patients who are new to ART as they are likely to struggle with adherence compared to those who have been on ART for a longer duration. Emerging evidence shows that strengthening psychosocial services and peer interaction through teen clubs improves ART adherence [48]. Therefore, providing resources to strengthen teen clubs is an important area that can improve ART adherence among newly disclosed adolescents as well as newly initiated treatment. Teen clubs are currently only available in facilities that are supported by non-governmental organization partners working with the ministry of health which means some ALHIV living in catchment areas not covered by these partners do not benefit from teen club services.

Availability of teen club services and friendly and supportive health workers greatly influence ART among adherence. This finding builds upon what was reported earlier in Malawi where adolescents attending teen clubs where psychosocial services were readily available registered improved ART adherence compared to those who have no access to such service [48]. The concept of youth-friendly services and adolescent health programming that promote teen clubs and psychosocial counseling as part of the ART package should be intensified. ALHIV are free to express themselves on adherence challenges they face when they find healthcare providers friendly and accommodative [4, 22, 55]. This calls for more training and investment in human resources and adequate training on youth-friendly services.

Although similar studies conducted in sub-Saharan Africa indicate a high pill burden as a barrier to ART adherence [9, 11, 56], our study did not find any significant change in ART adherence even after the addition of IPT to the ART package. Our study found that pill burden was problematic only in the first few days of starting IPT. This is in line with findings from earlier studies conducted in Malawi, Zimbabwe, and South Africa which found that ALHIV reports suboptimal adherence when a new intervention has been introduced due to fear of adverse effects and a lack of understanding [8, 57, 58]. Elsewhere, studies reported that ART adherence in the adolescence period is associated with multiple factors. Results from studies done in Europe, Asia, and Africa are consistent with our findings except on the aspect of pill-burden causing poor adherence[3, 5, 59–61]. The discrepancy may be a result of advanced services being offered at the study site. Among others, the routine adherence counseling, teen club sessions and recreation camps offered at BCMCFM-COE for ALHIV facilitate a good understanding of the disease and treatments being offered. These services have proven to be key interventions for improving ART adherence in ALHIV both in the United States of America and sub-Saharan Africa [6, 13, 14, 19, 20]. This finding further persuades policymakers to put much effort into empowering service providers with the necessary knowledge so that periodic client counseling should be part of lifelong therapy.

Despite acknowledging the importance of IPT in TB prevention, our study found that most ALHIV were non-adherent to IPT. Among others, the perception that IPT is an inferior drug, fear of side effects, added pill burden and poor pre-IPT initiation counseling were given as the reason for poor IPT adherence. This is in line with a Kenyan study done in children which reported that IPT related health education and counseling is crucial to IPT adherence and completion, while fear of adverse drug reaction and pill burden were the main barriers [62].This finding implores service providers to address any fears or misconceptions of new interventions by counseling and explaining with clarity the reason for a new intervention for improved acceptability.

### Strengths and limitations

The use of mixed methods design was a strength because it increased the richness of our data thereby enhancing study validity. Participant interviews helped us to learn more about the inner feelings ALHIV have concerning their treatment. The use of client records further improved our understanding of adherence trends following an intervention. One other key achievement of our study is that it has generated potential areas for policy intervention in HIV programming concerning ALHIV. This is especially an important achievement as it reflects on how ALHIV in resource-limited settings are likely to be affected by interventions being implemented by the ministry of health. Although the study provides vital insights into areas of improvement in the provision of care for ALHIV, it had some limitations. This study was conducted at a tertiary facility that is supported by external funds which may lead to participants having a biased view of HIV services. The presence of advanced services that are not routinely available in other facilities such as psycho-social support, may as well influence how ALHIV handle adherence challenges. Secondly, participants selected for review in the Pre-IPT review were largely not the same as those we selected for the post-IPT implementation review, which could lead to inaccurate inferences about the possible added pill burden. Additionally, since the study was only conducted at one site, it would be difficult to generalize these findings to all adolescents on ART in Malawi. The use of EMR which may be prone to data entry errors could also affect ART adherence calculations. Finally, the inclusion of self-reported ART adherence can lead to the problem of overestimation of a measure. However, these limitations were minimized by including participants with extreme adherence behaviors, analyzing records of participants who had at least 2 clinic visits during the study period, and cross-checking ART adherence with physical ART master cards or health passport books.

## Conclusion

Adolescents living with HIV face diverse, unique, and pressing challenges that affect optimal adherence to ART including pill burden. Evidence from this study demonstrates that the high pill burden as a result of IPT did not bring a significant pill burden that can potentially encumber optimal adherence. However, the study has overtly revealed that optimal adherence in ALHIV is promoted by other factors such as the presence of peer and parental support, pre-counseling of a new intervention, and use of medical recreation programs such as Teen clubs. These findings beseech the importance of targeting and strengthening the integration of youth-friendly services such as Teen clubs and recreational camps into HIV programming. It further implores policymakers to channel resources to training more health-workers on psychosocial counseling so that they can competently and confidently handle complex adherence issues that ALHIV face as they take ARVs. Further research should be done on a larger scale with the main focus on facilities that have limited psychosocial support to fully understand the factors that influence adherence among ALHIV.
